# Transcutaneous auricular vagus nerve stimulation on upper limb motor function with stroke: a functional near-infrared spectroscopy pilot study

**DOI:** 10.3389/fnins.2023.1297887

**Published:** 2023-11-21

**Authors:** Likai Wang, Fei Gao, Yongli Dai, Zhan Wang, Feng Liang, Jingyi Wu, Mengchun Wang, Litong Wang

**Affiliations:** ^1^Department of Rehabilitation Medicine, The Second Hospital of Dalian Medical University, Dalian, China; ^2^First Clinical Medical College, Shanxi Medical University, Taiyuan, China

**Keywords:** transcutaneous auricular vagal nerve stimulation, upper limb motor function, functional near-infrared spectroscopy, stroke, primary somatosensory cortex, pre-motor and supplementary motor cortex, primary motor cortex

## Abstract

**Background:**

Transcutaneous auricular vagus nerve stimulation (taVNS) emerges as a promising neuromodulatory technique. However, taVNS uses left ear stimulation in stroke survivors with either left or right hemiparesis. Understanding its influence on the cortical responses is pivotal for optimizing post-stroke rehabilitation protocols.

**Objective:**

The primary objective of this study was to elucidate the influence of taVNS on cortical responses in stroke patients presenting with either left or right hemiparesis and to discern its potential ramifications for upper limb rehabilitative processes.

**Methods:**

We employed functional near-infrared spectroscopy (fNIRS) to ascertain patterns of cerebral activation in stroke patients as they engaged in a “block transfer” task. Additionally, the Lateralization Index (LI) was utilized to quantify the lateralization dynamics of cerebral functions.

**Results:**

In patients exhibiting left-side hemiplegia, there was a notable increase in activation within the pre-motor and supplementary motor cortex (PMC-SMC) of the unaffected hemisphere as well as in the left Broca area. Conversely, those with right-side hemiplegia displayed heightened activation in the affected primary somatosensory cortex (PSC) region following treatment.

Significantly, taVNS markedly amplified cerebral activation, with a pronounced impact on the left motor cortical network across both cohorts. Intriguingly, the LI showcased consistency, suggesting a harmonized enhancement across both compromised and uncompromised cerebral regions.

**Conclusion:**

TaVNS can significantly bolster the activation within compromised cerebral territories, particularly within the left motor cortical domain, without destabilizing cerebral lateralization. TaVNS could play a pivotal role in enhancing upper limb functional restoration post-stroke through precise neuromodulatory and neuroplastic interventions.

## 1. Introduction

Stroke is a significant public health concern globally, with increasing incidence and prevalence rates annually (Prior and Suskin, 2018). The disability rate associated with stroke is alarmingly high, profoundly impacting the quality of life for patients. Upper limb dysfunction is among the most common and challenging sequelae of stroke. Recovery of the upper limb often lags behind that of the lower limb, especially in the restoration of fine motor skills, which can take considerably longer (Ghaziani et al., 2018). Consequently, innovative rehabilitation methods are essential to improve upper limb function and, by extension, the quality of life for stroke survivors.

In recent years, transcutaneous auricular vagus nerve stimulation (taVNS) has emerged as a promising non-invasive intervention for rehabilitating upper limb functional deficits post-stroke (Baig et al., 2022). This approach involves non-invasive stimulation of the auricular branch of the vagus nerve in the cymba conchae region, using a low-frequency pulse microcurrent device (Gianlorenco et al., 2022). The afferent signals from the stimulated auricular vagus nerve activate the nucleus tractus solitarius (NTS), exciting basal ganglia neurons and locus coeruleus neurons (Trevizol et al., 2015). This results in the release of neurotransmitters acetylcholine (ACh) and norepinephrine (NE), which widely activate the brain’s neuromodulatory network, enhancing neural plasticity (Morrison et al., 2021). While the vagus nerve stimulation causes the release of Ach and NE across the cortex, triggering cortical plasticity is a unique ability of a subset of neurons driven by sound or movement (Meyers et al., 2018; Collins et al., 2021). Changes in neurophysiology can be observed when the basal ganglia or vagus nerve is directly stimulated in a short timeframe accompanied by sound or movement. Studies indicate that combining taVNS with sensory or motor training can drive task-specific plasticity in the motor cortex (De Ridder et al., 2014; Baig et al., 2019). Compared to rehabilitation alone, synaptic connections in the corticospinal tract controlling the impaired forelimb increase, which is more conducive to limb function recovery (Hays et al., 2013). Moreover, combining vagus nerve stimulation with upper limb rehabilitation training can significantly improve limb function, especially if the training immediately follows the stimulation (Khodaparast et al., 2014, 2016). This synergistic effect between vagus nerve stimulation and rehabilitation appears to be time-dependent (Hays et al., 2016; Khodaparast et al., 2016).

The exact mechanisms by which taVNS influences cortical plasticity in the motor cortex remain elusive. Neural plasticity is foundational for motor recovery post-stroke. The balance between the adrenergic and cholinergic systems is key for taVNS to enhance neural plasticity (Morrison et al., 2021), yet the underlying neural mechanisms remain unclear. Two prevalent models of post-stroke functional recovery are the compensatory model and the interhemispheric competition model (Jaillard et al., 2005; Grefkes and Fink, 2014). The compensatory model (Jaillard et al., 2005; Small et al., 2013) suggests that brain regions outside the lesion, including the unaffected hemisphere, contribute to post-stroke functional recovery. In contrast, the interhemispheric competition model (Murase et al., 2004) posits that a stroke not only reduces the affected hemisphere’s inhibition of the unaffected hemisphere but also increases the latter’s inhibition of the former, causing a dual impediment in the affected hemisphere. However, these models might oversimplify and may not apply universally to all stroke patients. Di Pino and colleagues introduced the concept of structural preservation, leading to a bimodal balance-recovery model (Di Pino et al., 2014). According to this model, when structural preservation is high, the interhemispheric competition model is more predictive of recovery, whereas the compensatory model dominates when structural preservation is low. Recent evidence suggests that an upper limb Fugl-Meyer score of 43 can serve as a benchmark for structural preservation, distinguishing different degrees of upper limb functional impairment (Lin et al., 2020).

To delve deeper into the cortical activation patterns of stroke patients during upper limb movement control under taVNS, we conducted this study. We employed functional near-infrared spectroscopy (fNIRS) as our primary tool, allowing real-time monitoring of dynamic brain activity changes. Utilizing this method, we can observe cortical changes under taVNS stimulation, especially in brain regions associated with motor function recovery (Kunii et al., 2021; Zhang et al., 2023). This level of insight was previously unachievable with older methods. Task-based fNIRS paradigms offer a powerful tool for studying neural plasticity, providing a dynamic view of brain function (Pinti et al., 2020; Guo et al., 2022). However, standardized testing paradigms and technical indices for clinical applications are still in development, and many past studies have adjusted motion paradigms based on the specific circumstances of the patients in the study design (Urushidani et al., 2018; Lim and Eng, 2019).

This study aims to use fNIRS to monitor the brain activation effects of taVNS on stroke patients during upper limb transfer movements. We seek to explore the potential mechanisms of taVNS in upper limb rehabilitation and compare the differences in brain activation between patients with left-sided and right-sided hemiplegia.

## 2. Methods

### 2.1. Participants

A total of 27 stroke patients were enrolled in this randomized, single-blind, parallel-group pilot study. The participants were selected from stroke patients admitted to the Rehabilitation Medicine Department of the Second Affiliated Hospital of Dalian Medical University, China, between May and August 2023. Out of these patients, 15 had right-sided lesions and 12 had left-sided lesions. Table 1 presents the demographic and clinical characteristics of the participants. All participants were right-handed.

**Table 1:** Demographic and clinical characteristics of participants.

Number	Paralyzed side	Gender	Age	Stroke type	Lesion side	Lesion site	FMA-UE
001	Left side	Female	57	Ischemic stroke	Right side	Basal ganglia	35
002	Left side	Female	45	Ischemic stroke	Right side	Cerebral hemispheres	28
003	Left side	Female	58	Ischemic stroke	Right side	Corona radiata, Basal ganglia	22
004	Left side	Female	67	Ischemic stroke	Right side	Parietal occipital lobe	41
005	Left side	Female	77	Ischemic stroke	Right side	Corona radiata, Basal ganglia	30
006	Left side	Female	76	Ischemic stroke	Right side	Geniculate corpus callosum, Frontal lobe	23
007	Left side	Male	66	Ischemic stroke	Right side	Posterior limb of the internal capsule	37
008	Left side	Male	44	Ischemic stroke	Right side	Radiation crown, Basal ganglia	25
009	Left side	Male	59	Ischemic stroke	Right side	Frontotemporal parieto-occipital lobe, Basal ganglia, Lateral ventricles	29
010	Left side	Male	66	Ischemic stroke	Right side	Basal ganglia	32
011	Left side	Male	67	Intracerebral hemorrhage	Right side	Basal ganglia, Thalamus	38
012	Left side	Female	74	Ischemic stroke	Right side	Basal ganglia	27
013	Left side	Male	69	Ischemic stroke	Right side	Basal ganglia	21
014	Left side	Male	75	Ischemic stroke	Right side	Basal ganglia	40
015	Left side	Male	54	Ischemic stroke	Right side	Thalamus	34
016	Right side	Male	48	Intracerebral hemorrhage	Left side	Basal ganglia	24
017	Right side	Male	65	Ischemic stroke	Left side	Pons, Cerebellar hemisphere	42
018	Right side	Male	77	Ischemic stroke	Left side	Corona radiata	31
019	Right side	Male	61	Ischemic stroke	Left side	Lateral paraventricular	36
020	Right side	Female	71	Ischemic stroke	Left side	Pons	33
021	Right side	Male	52	Intracerebral hemorrhage	Left side	Parieto-occipital lobe	26
022	Right side	Male	64	Ischemic stroke	Left side	Cerebellar hemisphere	20
023	Right side	Male	42	Ischemic stroke	Left side	Frontotemporoparietal multifocal	39
024	Right side	Male	44	Ischemic stroke	Left side	Basal ganglia-radial crown	43
025	Right side	Female	76	Ischemic stroke	Left side	Lateral paraventricular	22
026	Right side	Male	73	Intracerebral hemorrhage	Left side	Basal ganglia	37
027	Right side	Male	54	Intracerebral hemorrhage	Left side	Basal ganglia, Cerebellar hemisphere	30

The patients were randomized using a computer-generated random sequence to determine their assignment into the two parallel groups: taVNS-left hemiplegia and taVNS-right hemiplegia. This randomization process ensured that each patient had an equal chance of being allocated to either of the study groups. As a single-blind study, the participants were unaware of their group assignments. However, the researchers and therapists administering the taVNS intervention were informed. The single-blind design was chosen to reduce potential biases in patient-reported outcomes and observations during the fNIRS sessions.

The inclusion criteria are as follows: (1) aged between 18 and 80 years, (2) newly diagnosed with either ischemic or hemorrhagic stroke according to the diagnostic criteria of cerebrovascular diseases in China (version 2019), (3) had a unilateral subacute or chronic stroke caused by the subcortical or cortical lesion (>3 weeks from stroke onset), (4) had scored at stage 3 or above on the Brunnstrom Upper Limb Motor Function assessment ensuring that participants demonstrated some degree of voluntary movement in the affected limb, and displayed motor dysfunction with a score of less than 43 on the Fugl-Meyer upper extremity assessment (FMA-UE), (5) able to cooperate with the assessment and taVNS intervention, (6) able to complete the fNIRS task paradigm for this study.

The exclusion criteria are as follows: (1) has severe diseases of the cardiovascular, digestive, or endocrine systems, (2) has other neurological or musculoskeletal disorders that might interfere with the study assessment, (3) has infections, ulcers, or scars on the auricle, (4) has the presence of metallic implants in the skull, hypersensitivity, injuries, or inflammations in the ear, (5) has a heart rate below 60 beats per minute, or the presence of devices like pacemakers or cochlear implants, (6) had previously underwent vagus nerve surgery, (7) unable to understand and follow instructions.

All patients provided signed informed consent and were informed of potential adverse events prior to the trial. This study protocol was approved by the Ethics Committee of the Second Affiliated Hospital of Dalian Medical University (approval number: 2023–058). The trial was registered with the China Clinical Trial Registration Center (registration number: ChiCTR2300069403).

### 2.2. Procedures

**Figure 1:**
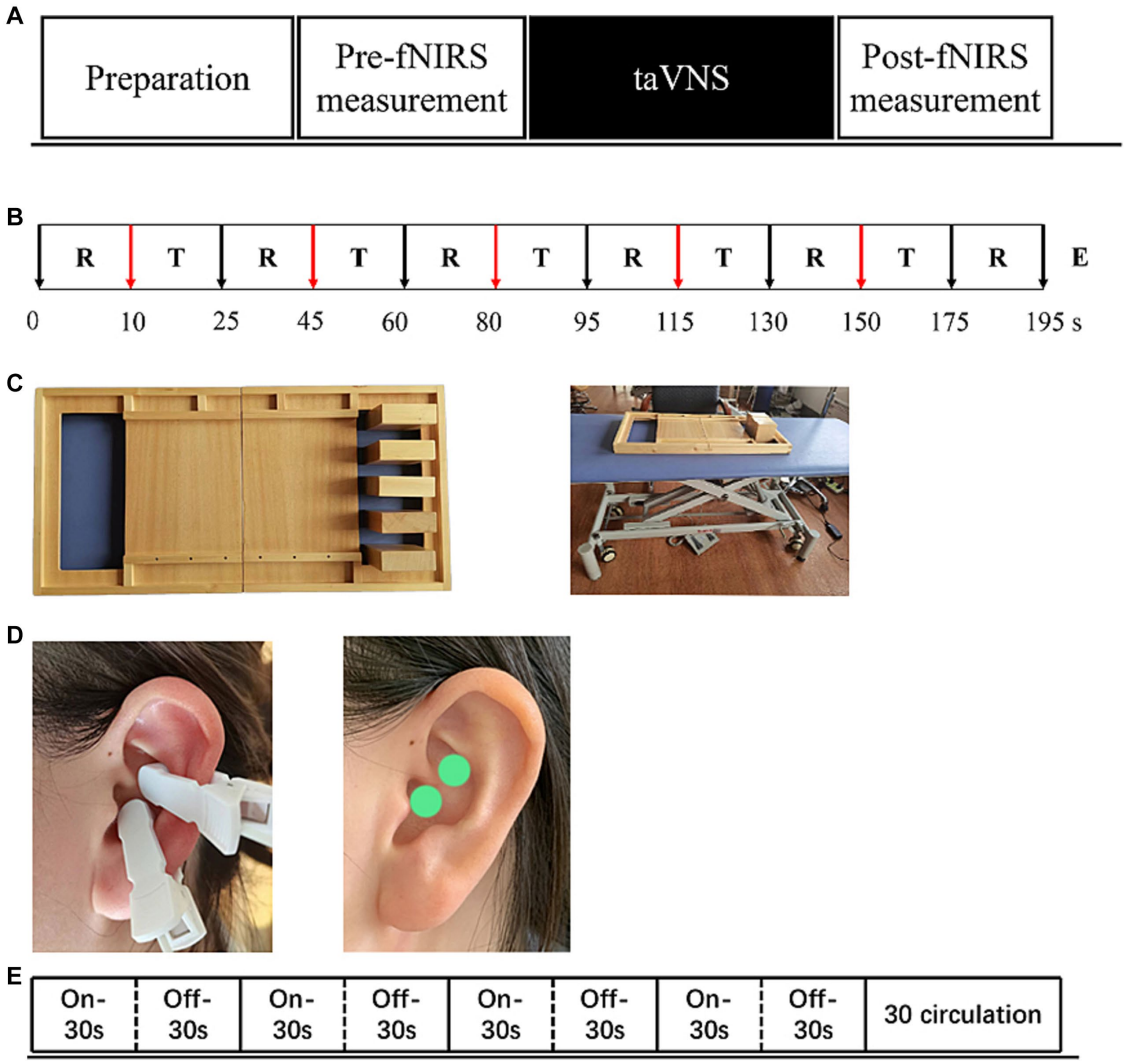
The experimental procedure **(A)** includes pre-measurement, taVNS intervention and post-measurement three parts. **(B)** “T”-Task, “R”-Rest, “E”-End. Show exact procedures of pre and post measurement and indicates task fNIRS measurement lasting for 5-min. **(C)** Indicates the stimulation mode of taVNS, a total of 30 cycles, each cycle, stimulation 30s, rest 30s.**(D)** Show the stimulus location of taVNS. **(E)** Supplies showing task paradigms - wooden blocks and adjustable tables and chairs.

Each patient recruited was first screened using FMA-UE and Brunnstorm to identify motor defects. All patients were randomized into two parallel groups-taVNS-left hemiplegia and taVNS-right hemiplegia. Subsequently, all patients underwent an initial fNIRS measurement while performing the block tasks but without any taVNS intervention (pre-fNIRS), as detailed in Figure 1B. After this initial measurement, patients received the taVNS intervention. Once the taVNS session concluded, another round of fNIRS measurement was conducted while the patients performed the block tasks, this time paired with the residual effects of the taVNS intervention (post-fNIRS), as shown in Figures 1A,B.

### 2.3. Functional near-infrared spectroscopy

**Figure 2:**
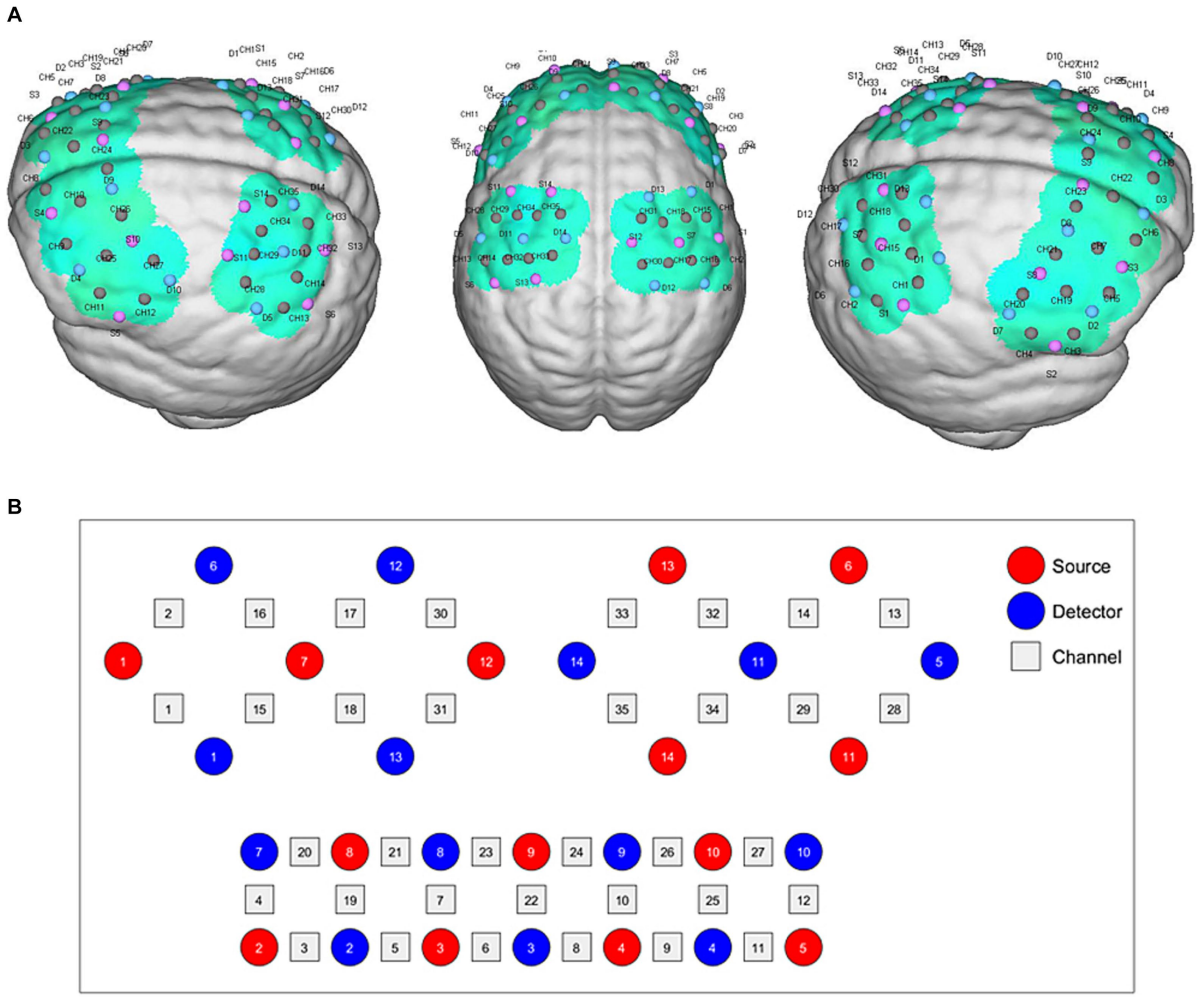
fNIRS montage. **(A)** Channels arrangements with numbers marked on a three-dimensional brain model in left and right vision. **(B)** Graphic design of each source, detector, and channel.

Functional near-infrared spectroscopy data were acquired using a 34-multichannel fNIRS instrument device (NirScan, Danyang Huichuang Medical Equipment Co., Ltd.,) with a sampling rate of 11 Hz. The wavelengths were set at 730 and 850 nm. Hemodynamic responses were recorded from the block-design task. The source and detector probe montage and cortical representation area are shown in Figure 2 and Table 2. The majority of the prefrontal, partial parietal, and occipital lobes were covered. The distance between the sources and detectors was 3 cm. The fNIRS recording was conducted by a trained therapist in a quiet specific treatment room. The fNIRS measurement was conducted before and immediately after the taVNS session. For clarity, the first fNIRS measurement (pre-fNIRS) was taken while participants engaged in the block tasks but without any taVNS. After undergoing taVNS, the second fNIRS measurement (post-fNIRS) captured neural activity while the participants repeated the block tasks, allowing for the assessment of taVNS-induced neural changes.

**Table 2:** The brain representative area on a Brodmann template under each channel (35 channels).

ROI	Region	Channels-right hemisphere	Channels-left hemisphere
Sensory network	1,2,3- Primary somatosensory cortex	2,16,17	13,14
Motor network	6 - Pre-motor and supplementary motor cortex	1,15,16,17,18,30,31	28,29,32,33,34,35
	4 - primary motor cortex	1,2,15,16,17,30	13,14,28,29,32,33
Wernicke network	40 - Supramarginal gyrus part of Wernicke’s area	2	13
Broca network	44 - pars opercularis part of Broca’s area	4,20	12,27
	45 - pars triangularis Broca’s area	3,4,19,20,21	11,12,25,27
DLPFC network	46 - Dorsolateral prefrontal cortex	3,5,7,19,20,21,23	9,10,11,25,26,27
	9 - Dorsolateral prefrontal cortex	21,23	10,24,26,27
Prefrontal	10 - Frontopolar area	5,6,7,23	8,9,10,24
	11 - Orbitofrontal area	6	

The Simplified Upper Limb Function Test (STEF) is designed to provide a rapid and straightforward assessment of upper limb motor skills. Given the neural plasticity induced by taVNS, we incorporated one specific task from STEF involving the lateral movement of wooden blocks. This task serves as a simulation of manual object retrieval commonly performed in daily activities. For the experimental setup, the STEF toolkit was employed along with height-adjustable armchairs to accommodate participants of varying heights. This setup is illustrated in Figure 1C. This minimizes the potential for interference arising from motor incoordination. In the task, participants are required to move five wooden blocks from one side of a square frame to the opposite side.

The experimental paradigm was structured in discrete blocks, each consisting of a 20-s rest period followed by a 15-s balancing task. Each full measurement cycle included a 10-s baseline measurement and five such blocks. Prior to task initiation, a 10-s resting-state data collection was performed, during which participants were instructed to sit quietly, refraining from physical movement and cognitive activity. The total duration of the task was 195 s. Before data collection, all participants underwent a training session to ensure they could perform the task accurately and without extraneous movements. Auditory cues were employed to signify the start and end points of each task phase. Initially, participants were seated in the adjustable armchair and instructed to remain still upon hearing the command “please rest.” They were then directed to move the blocks upon hearing either “move your left hand” or “move your right hand,” and to return to a resting state upon hearing “please rest” again. Participants were specifically instructed to abstain from speaking or executing additional head movements throughout the task.

### 2.4. Transcutaneous auricular vagus nerve stimulation

TaVNS therapy is administered using a low-frequency pulse electrostimulator, provided by Shanghai Xibei Electronic Technology Development Co., Ltd., with the model designation of En-stim4. The device is equipped with two circular metal electrodes, each measuring 5 × 5 mm in diameter. Prior to application, the auricular region of the left ear is cleansed using alcohol and allowed to dry. The electrodes are then positioned on the cymba conchae region for the delivery of electrical stimulation, as depicted in Figure 1D.

Stimulation parameters are set with a frequency and pulse width of 25 Hz and 300 μs, respectively. Each stimulation phase lasts for 30 s, followed by a 30-s pause as shown in Figure 1E. Biphasic sinusoidal pulses are employed throughout the therapy session, which has a total duration of 30 min. The stimulation intensity starts at the lowest level and is gradually increased until the patient experiences pain. The final current is then adjusted to just below the pain threshold.

### 2.5. fNIRS data processing

#### 2.5.1. Data analysis

The processing of task-related fNIRS data was executed using the NirSpark software toolkit. The data were preprocessed by setting the standard deviation of the signal at a threshold value of 6 and the peak threshold at 0.5. Motion artifacts were identified and removed through spline interpolation methods. To filter out general noise, including cardiac, respiratory, and Mayer waves, a bandpass filter with a frequency range of 0.01–0.1 Hz was applied. The path-length differential factors were set between −6 and 6, and real-time concentration changes of oxyhemoglobin (HbO2) and deoxyhemoglobin (HbR) during the tasks were calculated based on the modified Beer–Lambert Law.

#### 2.5.2. Task-related cortical activation analysis

The BlockAvg module within NirSpark was utilized for block averaging analysis and linear correction of the block design task involving object manipulation. An average was computed for the block tasks repeated five times, resulting in a 35-s task block (the first 15 s for the object manipulation task and the subsequent 20 s for rest). A General Linear Model (GLM) was employed to examine the correlation between blood oxygen level changes and the temporal sequencing of tasks. Hemodynamic response functions (HRFs) were used as basis functions for the design matrix. Baseline drift was removed, and short-term correlations associated with cardiac and respiratory high-frequency noise were corrected. The developed design matrix was then fitted to the collected data. For data adhering to a normal distribution, paired-sample *t*-tests were conducted, whereas the non-parametric Wilcoxon signed-rank test was used for data not normally distributed. A value of *p* less than 0.05 was considered statistically significant and served as the criterion for determining whether significant differences existed in the *β* indices before and after taVNS intervention. Two-dimensional channel-wise visualization was used to display group-level fNIRS analysis indices (*β* values or contrast values).

#### 2.5.3. Laterality index

The Laterality Index (LI) was employed to assess the asymmetry of activation across different regions (Vingerhoets et al., 2023). Feature analysis was carried out on the mean value in the 15-s task interval post block-averaging. The oxygen concentration changes in individual channels during this time window were calculated. The LI was defined as [Δoxy-Hb in the affected hemisphere – Δoxy-Hb in the unaffected hemisphere]/[Δoxy-Hb in the affected hemisphere + Δoxy-Hb in the unaffected hemisphere] (Ohyanagi et al., 2018). It is generally considered that an LI greater than or equal to 0.1 indicates lesional-side lateralization, while an LI less than or equal to −0.1 suggests healthy-side lateralization (Vernooij et al., 2007).

## 3. Results

The clinical characteristics of the two groups are shown in Table 3. Differences were not observed in the patient’s demographic characteristics including age, gender, course of a stroke, FMA-UE, and primary diagnosis between the two groups in the baseline. All patients completed the intervention and measurement process, and no adverse events happened in all groups.

**Table 3:** Baseline characteristics of the two patients’ groups.

	Group-left hemiplegia (*n* = 15)	Group-right hemiplegia (*n* = 12)	*p*-value
Age (years)	63.6 ± 10.53	60.58 ± 12.42	0.501^a^
Gender (male/female)	8/7	10/2	0.218^b^
Course of stroke (days)	25.0 (10.5)	32.5 (46.75)	0.365^c^
FMA-UE	30.8 ± 6.56	31.92 ± 7.75	0.689^a^
Primary diagnosis			0.203^b^
Hemorrhagic	1	4	
Ischemic	14	8	
Underlying diseases			0.759^b^
Hypertension	12	9	
Diabetes mellitus	5	4	
Other conditions	2	1	

Data are expressed as Mean ± SD, *N* (%), or Mid (IQR).

^a^one way ANOVA; ^b^Chi-Square test; ^c^Kruskal-Wallis test.

### 3.1. Cortical mapping of hemiplegic upper limb transfer movements

**Figure 3:**
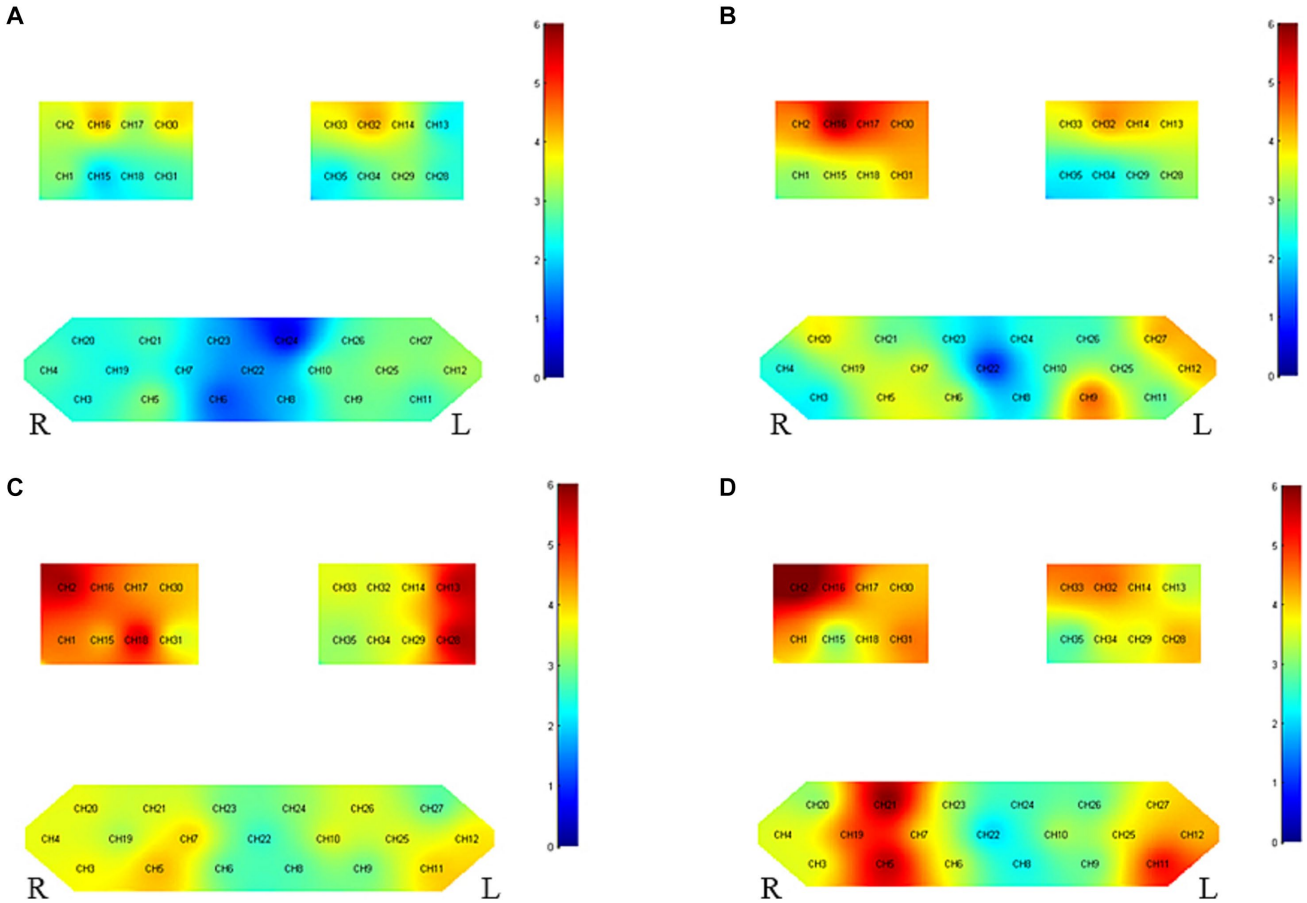
Group-level channel activation *t*-map in the task (*p* < 0.05, uncorrected). **(A)** Grasping wooden blocks before intervention for right hemiplegia **(B)** grasping wooden blocks after right hemiplegia intervention, **(C)** grasping wooden blocks before intervention for left hemiplegia and **(D)** grasping wooden blocks after left hemiplegia intervention.

Figure 3 and Table 4 presents the trends of hemodynamic changes in oxy-Hb and deoxy-Hb concentrations across all channels under two conditions. This activation characteristic manifested a specific pattern both pre and post-intervention.

**Table 4:** List of group-level channel activation in the task (*t*-value, *p* < 0.05).

		Left hemisphere	Right hemisphere
Right hemiplegia	Before	CH9(2.853, 0.016), CH10(3.248, 0.008), CH11(2.464, 0.031), CH12(3.310, 0.007), CH14(3.691, 0.004), CH25(3.188, 0.009), CH26(2.724, 0.020), CH27(2.934, 0.014), CH28(2.313, 0.041), CH29(3.309, 0.007), CH32(4.795, 0.001), CH33(3.630, 0.004)**, CH34(2.695, 0.021)**	CH1(3.304, 0.007), CH2(3.260, 0.008)**, CH3(2.334, 0.040)**, CH4(2.786, 0.018), CH5(3.353, 0.006), CH16(4.803, 0.001), CH17(2.820, 0.017), CH18(2.398, 0.035), CH20(2.366, 0.037), CH21(2.790, 0.018), CH30(4.212, 0.001), CH31(2.589, 0.025)
Right hemiplegia	After	CH9(5.255, 0.000), CH10(2.823, 0.017), CH11(2.548, 0.027), CH12(4.340, 0.001)**, CH13(3.697, 0.004)**, CH14(4.215, 0.001)**, CH24(2.631, 0.023)**, CH25(2.542, 0.027), CH26(2.404, 0.035), CH27(4.440, 0.001), CH28(3.258, 0.008), CH29(2.508, 0.029), CH32(4.916, 0.000), CH33(3.570, 0.004)	CH1(2.905, 0.014), CH2(4.175, 0.002), CH4(2.314, 0.041), CH5(3.647, 0.004)**, CH6(3.611, 0.004), CH7(3.746, 0.003)**, CH15(3.257, 0.008), CH16(6.756, 0.000), CH17(5.027, 0.000), CH18(3.235, 0.008)**, CH19(3.578, 0.004)**, CH20(4.053, 0.002), CH21(2.656, 0.022)**, CH23(2.206, 0.050)**, CH30(4.331, 0.001), CH31(4.312, 0.001)
Left hemiplegia	Before	CH8(2.653, 0.019), CH9(2.694, 0.017), CH10(3.817, 0.002), CH11(4.132, 0.001), CH12(3.967, 0.001), CH13(6.085, 0.000), CH14(3.488, 0.004), CH24(3.074, 0.008), CH25(3.654, 0.003), CH26(3.556, 0.003), CH27(2.464, 0.027), CH28(6.209, 0.000), CH29(3.236, 0.006), CH32(3.404, 0.004), CH33(3.672, 0.003), CH34(3.481, 0.004), CH35(3.037, 0.009)	CH1(4.599, 0.000), CH2(6.107, 0.000), CH3(3.780, 0.002), CH4(3.586, 0.003), CH5(4.315, 0.001), CH6(2.603, 0.021), CH7(4.335, 0.001), CH15(3.650, 0.003), CH16(4.680, 0.000), CH17(4.191, 0.001), CH18(6.453, 0.000), CH19(2.995, 0.010), CH20(3.672, 0.003), CH21(3.436, 0.004)**, CH22(2.362, 0.033)**, CH23(2.823, 0.014), CH30(4.067, 0.001), CH31(2.996, 0.010)
Left hemiplegia	After	CH8(2.223, 0.043), CH9(2.681, 0.018), CH10(3.531, 0.003), CH11(5.543, 0.000), CH12(4.065, 0.001), CH13(3.092, 0.008), CH14(4.116, 0.001), CH24(2.649, 0.019), CH25(3.820, 0.002), CH26(2.550, 0.023), CH27(3.904, 0.002), CH28(4.379, 0.001), CH29(3.380, 0.004), CH32(4.947, 0.000), CH33(4.602, 0.000), CH34(3.764, 0.002), CH35(2.350, 0.034)	CH1(4.460, 0.001), CH2(6.825, 0.000), CH3(3.424, 0.004), CH4(3.635, 0.003), CH5(6.024, 0.000), CH6(3.605, 0.003), CH7(3.583, 0.003), CH15(2.235, 0.042), CH16(5.869, 0.000), CH17(3.780, 0.002), CH18(3.974, 0.001), CH19(5.046, 0.000), CH20(2.763, 0.015), CH21(6.495, 0.000), CH23(3.165, 0.007), CH30(4.058, 0.001), CH31(4.739, 0.000)

The meaning of the bold values represents the different channels in the activated channels before and after intervention.

#### 3.1.1. Cortical activation in patients with left-side hemiplegia

Before intervention, there was pronounced bilateral brain cortical activation (35/35 channels), especially in the motor cortex. Notable activation was observed in the affected side’s primary somatosensory cortex (PSC) (CH2), pre-motor and supplementary motor cortex (PMC-SMC) (CH1/15/18), and primary motor cortex (M1) (CH16/17/30), as compared to the unaffected side’s PSC (CH13) and PMC-SMC (CH28). Activity was also seen in the left Broca area (CH11/12) and Frontopolar area (CH5/7). After intervention, bilateral activation remained stable (34/35 channels), especially in the motor cortex. Significant activation was noted in the affected side’s PSC (CH2), PMC-SMC (CH1/18/31), and M1 (CH16/17/30) and the unaffected side’s M1 (CH14/32) and PMC-SMC (CH28/29/33/34).

#### 3.1.2. Cortical activation patterns in patients with right-side hemiplegia

Before intervention, activation was present in certain channels, especially in the affected brain’s M1 (CH14/32) and PMC-SMC (CH33), and the unaffected brain’s M1 (CH16/30). After intervention, the affected side’s M1 (CH14/32), PMC-SMC (CH33), and PSC (CH13) exhibited significant activation. The unaffected side’s PSC (CH2), PMC-SMC (CH31), M1 (CH16/17/30), Frontopolar area (CH9), and left Broca area (CH12/27) all showed notable activity.

#### 3.1.3. Comparison of activation between left and right hemiplegic patients

Before intervention, bilateral brain cortical activation was more pronounced in patients with left-side hemiplegia (35/35 channels) than in those with right-side hemiplegia (25/35 channels). Activation for both groups was mainly localized in the motor cortex. After intervention, the activation region was broader and more intense in patients with left-side hemiplegia (34/35 channels) compared to those with right-side hemiplegia (30/35 channels).

#### 3.1.4. Inter-group differences post-treatment

**Figure 4:**
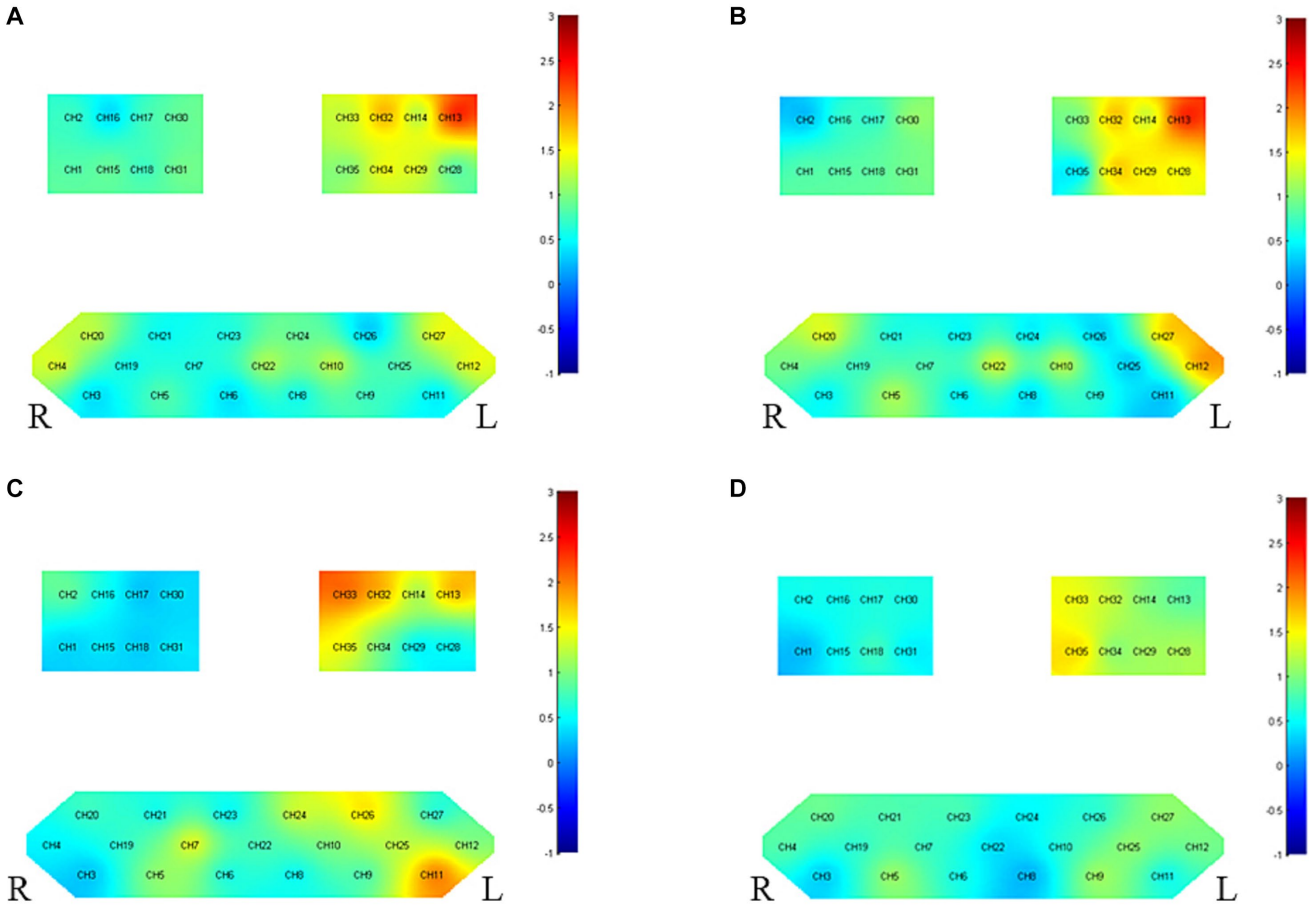
Between-group variance of channel activation *t*-map in the task (*p* < 0.05, uncorrected). **(A)** The variance of *β* before and after intervention for right hemiplegia. **(B)** The variance of HbO2 before and after intervention for right hemiplegia. **(C)** The variance of *β* before and after intervention for left hemiplegia. **(D)** The variance of HbO2 before and after intervention for left hemiplegia.

Refer to Figure 4 and Table 5 for post-treatment inter-group differences. Enhanced activation was observed in the unaffected side’s PMC-SMC (CH33) and the left Broca area (CH11) in patients with left-side hemiplegia, but there was no significant change in average HbO2. For patients with right-side hemiplegia, stronger activation was displayed in PSC (CH13) for both *β* and HbO2 post-treatment. The current findings support the notion that taVNS does not directly enhance M1 activity.

**Table 5:** List of group-level channel activation in the task (*t*-value, *p* < 0.05).

Group	Index	Left hemisphere	Right hemisphere
Left hemiplegia before and after intervention	β	CH11(2.233,0.042) CJH33(−2.203,0.045)	None
	HbO2	None	None
Right hemiplegia before and after intervention	β	CH13(−2.725,0.020)	None
	HbO2	CH13(−2.682,0.021)	None

### 3.2. Assessment of cortical activation symmetry

**Figure 5:**
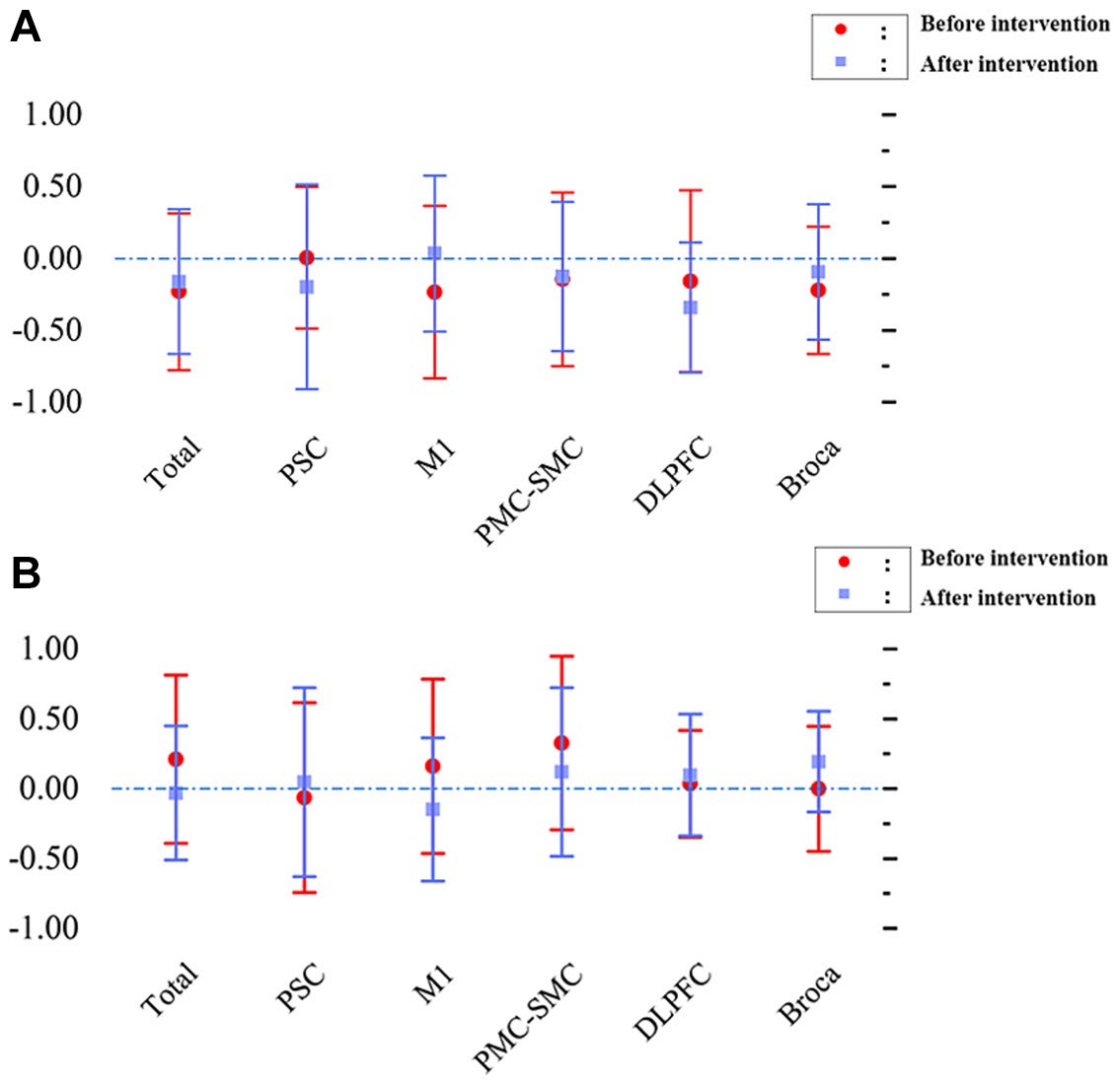
Changes in interhemispheric regional activation asymmetry during block transfer task in the two groups, as measured by LI. **(A)** L1 - patients with left hemiplegia before and after intervention. **(B)** L1 - patients with right hemiplegia before and after intervention.

Figure 5, displaying the LI, represents the changes in interhemispheric asymmetry of regional activation during the block transfer task among the groups.

#### 3.2.1. Patients with left hemiplegia

Before intervention, the LI values (mean ± SD) were 0.004 ± 0.492 in the PSC, −0.23 ± 0.600 in the PMC, −0.145 ± 0.603 in the SMC, −0.158 ± 0.633 in the DLPFC, and − 0.221 ± 0.443 in the Broca area. The overall value was −0.231 ± 0.546 for patients with left hemiplegia. A comprehensive analysis indicates that the LI for most brain regions was < −0.1, suggesting that during the block transfer task, activation was primarily concentrated in the affected brain region (right hemisphere). After intervention, the LI values (mean ± SD) were − 0.198 ± 0.713 in the PSC, 0.034 ± 0.542 in the PMC, −0.127 ± 0.519 in the SMC, −0.342 ± 0.452 in the DLPFC, and − 0.095 ± 0.472 in the Broca area. The overall value was −0.161 ± 0.503 for patients with left hemiplegia. The results showed that the LI for most brain regions was less than −0.1, indicating that activation in the affected brain region (right hemisphere) remained significant.

#### 3.2.2. Patients with right hemiplegia

Before intervention, the LI values (mean ± SD) were − 0.064 ± 0.678 in the PSC, 0.159 ± 0.624 in the PMC, 0.326 ± 0.620 in the SMC, 0.035 ± 0.381 in the DLPFC, and − 0.000 ± 0.447 in the Broca area. The overall value was 0.211 ± 0.601 for patients with right hemiplegia. Most brain regions had a LI > 0.1, with the primary activation being in the affected brain area (left hemisphere). After intervention, the LI values (mean ± SD) were 0.047 ± 0.677 in the PSC, −0.149 ± 0.512 in the PMC, 0.120 ± 0.603 in the SMC, 0.097 ± 0.436 in the DLPFC, and 0.194 ± 0.359 in the Broca area. The overall value was −0.031 ± 0.480 for patients with right hemiplegia. Similar to before the intervention, the LI for most brain regions was >0.1, indicating that the primary activation remained in the affected brain area (left hemisphere).

#### 3.2.3. Statistical analysis

For patients with left-side hemiplegia, repeated-measures ANOVA for LI indicated no statistically significant interaction for time factor (pre/post-intervention) x cortical area (F[5, 167] = 0.829, *p* = 0.531) or main effects (time factor: F[1, 167] = 0.039, *p* = 0.844; region factor: F[5, 167] = 0.349, *p* = 0.883). However, post-hoc tests revealed significant differences pre-intervention, like the overall versus PMC-SMC (*p* = 0.0201), which disappeared post-intervention, suggesting a need for more cortical recruitment in the non-dominant hemisphere that normalized after the intervention. For patients with right-side hemiplegia, such differences were neither seen pre-treatment nor post-treatment, indicating no similar differences in the dominant hemisphere.

For patients with right-side hemiplegia, the repeated-measures ANOVA for LI showed no significant interaction for the time factor × cortical area (F[5, 131] = 0.912, *p* = 0.475) or main effects (time factor: F[1, 131] = 0.511, *p* = 0.476; region factor: F[5, 131] = 0.559, *p* = 0.731). Post-hoc tests indicated no significant differences between all combinations pre and post-intervention (all *p* values >0.05).

In summary, these results suggest that when patients’ lateralization primarily leans towards the affected brain area, taVNS enhances activation channels without significantly impacting the LI, maintaining a beneficial brain activation pattern for hemiplegic patients.

## 4. Discussion

TaVNS has emerged as a promising non-invasive neuromodulatory intervention in the realm of stroke rehabilitation, particularly for upper limb dysfunction. Recent systematic reviews and network meta-analyses (Ahmed et al., 2022, 2023) consistently highlight the efficacy of taVNS in enhancing upper limb motor function and performance in activities of daily living post-stroke, both in acute/sub-acute and chronic phases. Notably, taVNS not only demonstrates potential benefits comparable to other non-invasive brain stimulation techniques but also appears to outperform traditional VNS in some aspects. Furthermore, the initial findings on its safety profile are encouraging (Dawson et al., 2016; Capone et al., 2017; Wu et al., 2020). Despite these promising results, the call for larger randomized controlled trials remains, aiming to refine the optimal stimulation paradigms and further establish the relative superiority of taVNS in the landscape of stroke rehabilitation (Baig et al., 2022; Ahmed et al., 2023). Additionally, investigating the neural effects of taVNS on the affected brain regions in stroke patients holds significant academic and clinical importance (Li et al., 2022). To our knowledge, this study is the first to explore the impact of taVNS on brain function in stroke patients with upper limb functional impairments, offering conclusive evidence for the same. Our research endeavors to provide valuable insights into the potential effects and practicality of the intervention, serving as a reference for subsequent research.

For patients with left hemiparesis, there is noticeable activation in both cerebral hemispheres. This could be attributed to their reliance on the undamaged right hemisphere to compensate and aid upper limb movements. Notably, core motor control regions such as the PSC, PMC-SMC, and M1 all exhibit significant activation, underscoring their pivotal roles in motor control and recovery. In contrast, patients with right hemiparesis display significant activation in the PMC-SMC and M1 regions of the impaired left hemisphere prior to intervention. The unaffected hemisphere displays more extensive activation post-treatment, suggesting that it might play a compensatory role in the recovery process. A plausible explanation for these observations is that a stroke might cause damage to the motor cortex and its descending corticospinal tracts, leading to muscle weakness (Grefkes and Fink, 2020). Following a stroke, patients might necessitate increased activation of motor cortical networks for upper limb motor control (Lim and Eng, 2019). Furthermore, when faced with intricate upper limb motor tasks, there’s even compensatory activation observed in the unaffected hemisphere (Li et al., 2020).When comparing left and right hemiparetic patients, those with left hemiparesis (right hemisphere damage) consistently exhibit more cortical activation both before and after interventions compared to those with right hemiparesis. This might imply that the efficiency of the non-dominant hemisphere in certain tasks could inherently be lower than that of the dominant hemisphere (Wang et al., 2023). It further suggests that, compared to right hemiparetic patients, those with left hemiparesis might need to recruit more brain regions to achieve similar levels of functional recovery.

TaVNS demonstrates differential cortical activation effects in patients with left versus right hemiparesis. In patients with left hemiparesis executing complex tasks, taVNS significantly augmented the compensatory activation in the unaffected (left) PMC-SMC region and the Broca’s area through channels CH33 (S13-D14) and CH11 (S4-D5). While differences in the left PSC brain region CH13 (S6-D5) and the M1 brain region CH32 (S13-D11) were not statistically significant, their values of *p* of 0.063 and 0.070, respectively, indicate a trend. The mean changes in HbO2 were not significant, but this could be related to the choice of measurement method, time window, or statistical methodology. Additionally, other neuroregulatory or neuroplasticity mechanisms might influence these outcomes. The PMC-SMC plays a central role in upper limb activity; the PMC primarily oversees prediction and planning of movements, while the SMC orchestrates complex movement sequences and patterns (Dey et al., 2023). Given the fine motor and coordination capabilities of the hand (Nachev et al., 2008), these regions ensure that hand movements are purposeful and intentional. The Broca’s area plays a critical role in various tasks, especially those involving complex hand-eye coordination and planning. In upper limb actions, Broca’s area may be involved in planning and directing intricate gestures and movements, particularly those associated with language or symbolic encoding (Sprung-Much et al., 2022). In the execution of upper limb movements, Broca’s area is postulated to relay signals to both the PMC and SMC to orchestrate and steer associated motor actions (Zaccarella et al., 2021). A robust interplay and connectivity among these regions are pivotal in ensuring the seamless integration of such movements. For patients with right upper limb impairment, taVNS significantly boosted the activation of the impaired side’s PSC cortex through channel CH13 (S6-D5). Interestingly, the difference in the M1 brain region CH32 (S13-D11) was not statistically significant, but its value of p of 0.061 also suggests a trend. This enhanced PSC activation could imply that taVNS has a more significant effect on the recovery of patients with right hemiparesis, correlating with the PSC’s role in sensory integration, spatial perception, and attention function restoration. The PSC mainly handles sensory inputs from various body parts, particularly during upper limb activities, where it’s the primary region receiving signals from tactile receptors in the hand and arm (Greig et al., 2016). These signals provide feedback about touch, pressure, temperature, and body position to the brain, ensuring accurate sensory feedback during upper limb tasks, thus optimizing movement. While the M1 plays a critical role in motor recovery and balancing functions, being fundamental in motor rehabilitation, the current results do not seem to support the notion that taVNS directly augments the M1 (Vaz et al., 2019; Kang et al., 2020). This could be related to the sample size or might suggest that taVNS effects are more through indirect influence on other brain regions rather than a direct action on the M1.

The LI is a quantitative measure specifically used to gauge the lateralization of brain function, especially in brain region activation patterns associated with particular tasks (Vingerhoets et al., 2023). taVNS has been observed to enhance the activation of specific brain regions in patients, possibly reflecting the neuromodulatory effects of taVNS. By promoting neural transmission and bolstering regional function, taVNS may aid the patient’s recovery process. However, notably, even though there was an increase in brain activation, the LI did not exhibit significant changes. This suggests that while taVNS elevates brain activity, this enhancement is balanced between the affected and unaffected brain regions, ensuring the stability of lateralization. Such stability might be viewed positively as it hints that taVNS does not lead to overstimulation in any brain region, thus minimizing potential adverse reactions or risks of excessive stimulation. Furthermore, these results underscore the importance of the LI in the recovery process from a stroke, serving as a pivotal tool to evaluate treatment efficacy and brain functional reorganization. In summary, these findings emphasize the potential application of taVNS in stroke rehabilitation, especially for those patients where lateralization predominantly tilts towards the affected brain region. While it can boost the activation of the impaired brain region, its limited impact on the LI might be pivotal to its safety and efficacy.

The research revealed that regardless of left or right hemiparesis, taVNS primarily enhanced the activation of the left motor cortical network. Earlier studies had revealed that compared to solely undergoing rehabilitation training, the combination of vagus nerve stimulation with upper limb rehabilitation training could markedly improve limb function in patients. Rong et al. unveiled that taVNS at 25 Hz can activate multiple brain regions, such as the precentral gyrus, contralateral postcentral gyrus, bilateral insula, and the prefrontal cortex (Wang et al., 2021). Additionally, research by Badran et al. (2022) also confirmed that taVNS can increase blood flow in various brain regions. In this study, we employed the fNIRS technique and selected the “block transfer” as the task paradigm. This choice was made based on the characteristics of fNIRS in upper limb tasks among stroke patients, where measurement errors are negatively correlated with motor activation (Zhou et al., 2022). This implies that stronger motor activation results in smaller measurement errors. Compared to the simple hand grasp task chosen by many other studies, the “block transfer” task, though more complex, is more common in daily life and requires a certain level of proficiency. When performing simple tasks like repetitive grasping, the brain primarily relies on subcortical structures without excessively activating the cortical motor areas. However, for more complex tasks, cortical activation is significantly enhanced, leading to bilateral cortical activation. Given the neural plasticity demonstrated by taVNS in stroke rehabilitation, choosing a task that’s both relevant to daily life skills and comparatively complex is crucial to maximizing the observation of taVNS’s training effects (Hays et al., 2016; Khodaparast et al., 2016).

In stroke rehabilitation treatment, how to apply taVNS on an individualized basis remains a challenge yet to be addressed. Although both left and right hemiparesis patients showed enhanced activation in the left brain regions, there are still significant differences in their responses to individual rehabilitation training. This could be due to the multitude of factors influencing individual recovery in upper limb motor tasks. Individual-level asymmetric activation is a prominent feature, and lateralization varies depending on the recovery stage. In this study, the patients had Fugl-Meyer upper extremity assessment scores less than 43, indicating that they belong to the category of patients with a lower degree of structural preservation. Hence, we preliminarily infer that taVNS might be more suitable for this specific patient group.

## 5. Limitations

This study has several limitations that provide further directions for future exploration. Firstly, the sample size was small, which was determined primarily based on practical considerations. We aimed for a sample that our resources could accommodate efficiently within our budget constraints. As this was a pilot study, our main objective was to gather preliminary data and evaluate the feasibility of the intervention. Future studies with a larger sample are needed to derive more conclusive results. In addition, due to methodological limitations, although the experiment mainly focused on the effects of taVNS on the cortex, future research should delve deeper into the interactions within the cortex and between the cortex and subcortical structures due to taVNS. Furthermore, it is crucial to recognize the inherent limitations of fNIRS as a neuroimaging tool. While fNIRS is adept at capturing cortical activity, its ability to capture deep brain activities, especially in subcortical regions, is constrained. Then, a limitation to note is the fNIRS measurement timing. Although we implemented a pre-fNIRS measurement during task performance without taVNS and a post-fNIRS measurement post-taVNS during task performance, external factors like the repetition of task performance might influence observed changes. Future research could consider more frequent fNIRS measurements or control tasks to discern the specific effects of taVNS more accurately. Lastly, to ensure the safety of the treatment, we used clinically common settings. Future studies should delve into the therapeutic outcomes of right-sided and bilateral stimulation, elucidating the relationship between stimulation sites and stroke lesion locations.

## 6. Conclusion

TaVNS can significantly bolster the activation within compromised cerebral territories, particularly within the left motor cortical domain, without destabilizing cerebral lateralization. TaVNS could play a pivotal role in enhancing upper limb functional restoration post-stroke through precise neuromodulatory and neuroplastic interventions.

## Data availability statement

The raw data supporting the conclusions of this article will be made available by the authors, without undue reservation.

## Ethics statement

The studies involving humans were approved by Ethics Committee of the Second Affiliated Hospital of Dalian Medical University. The studies were conducted in accordance with the local legislation and institutional requirements. The participants provided their written informed consent to participate in this study.

## Author contributions

LikW: Conceptualization, Data curation, Investigation, Methodology, Supervision, Writing – original draft, Writing – review & editing. FG: Conceptualization, Data curation, Writing – original draft, Writing – review & editing. YD: Investigation, Writing – original draft. ZW: Conceptualization, Formal analysis, Methodology, Writing – review & editing. FL: Methodology, Writing – review & editing. JW: Investigation, Writing – review & editing. MW: Formal analysis, Writing – review & editing. LitW: Conceptualization, Funding acquisition, Investigation, Methodology, Writing – original draft, Writing – review & editing.
